# Early supported discharge after stroke in Bergen (ESD Stroke Bergen): three and six months results of a randomised controlled trial comparing two early supported discharge schemes with treatment as usual

**DOI:** 10.1186/s12883-014-0239-3

**Published:** 2014-12-21

**Authors:** Håkon Hofstad, Bente Elisabeth Bassøe Gjelsvik, Halvor Næss, Geir Egil Eide, Jan Sture Skouen

**Affiliations:** Department of Physical Medicine and Rehabilitation, Haukeland University Hospital, PO Box 1400, Bergen, N-5021 Norway; Department of Global Public Health and Primary Care, Physiotherapy Research Group, University of Bergen, Bergen, Norway; Department of Physiotherapy, Haukeland University Hospital, Bergen, Norway; Department of Neurology, Haukeland University Hospital, Bergen, Norway; Centre for Age-related Medicine, Stavanger University Hospital, Stavanger, Norway; Institute of Clinical Medicine, University of Bergen, Bergen, Norway; Centre for Clinical Research, Haukeland University Hospital, Bergen, Norway; Department of Global Public Health and Primary Care, Lifestyle Epidemiology Research Group, University of Bergen, Bergen, Norway

**Keywords:** Stroke, Randomised controlled trial, Rehabilitation, Early supported discharge, ESD, Community rehabilitation

## Abstract

**Background:**

Stroke causes lasting disability and the burden of stroke is expected to increase substantially during the next decades. Optimal rehabilitation is therefore mandatory. Early supported discharge (ESD) has previously shown beneficial, but all major studies were carried out more than ten years ago. We wanted to implement and study the results of ESD in our community today with comparisons between ESD and treatment as usual, as well as between two different ESD models.

**Methods:**

Patients with acute stroke were included during a three year period (2008–11) in a randomised controlled study comparing two different ESD models to treatment as usual. The two ESD models differed by the location of treatment: either in a day unit or in the patients’ homes. Patients in the ESD groups were followed by a multi-disciplinary ambulatory team in the stroke unit and discharged home as early as possible. The ESD models also comprised treatment by a multi-disciplinary community health team for up to five weeks and follow-up controls after 3 and 6 months. Primary outcome was modified Rankin Scale (mRS) at six months.

**Results:**

Three-hundred-and-six patients were included. mRS scores and change scores were non-significantly better in the two ESD groups at 3 and 6 months. Within-group improvement from baseline to 3 months was significant in the ESD 1 (p = 0.042) and ESD 2 (p = 0.001) groups, but not in the controls. More patients in the pooled ESD groups were independent at 3 (p = 0.086) and 6 months (p = 0.122) compared to controls and there also was a significant difference in 3 month change score between them (p = 0.049). There were no differences between the two ESD groups. Length of stay in the stroke unit was 11 days in all groups.

**Conclusions:**

Patients in the ESD groups tended to be more independent than controls at 3 and 6 months, but no clear statistically significant differences were found. The added effect of supported discharge and improved follow-up seems to be rather modest. The improved stroke treatment of today may necessitate larger patient samples to demonstrate additional benefit of ESD.

**Clinical trial registration:**

Unique identifier: NCT00771771

## Background

Stroke is the most common cause of lasting disability in older age, leading to both personal and familial burdens as well as high societal costs [1,2]. The increasing incidence of stroke with higher age and decreasing stroke mortality [3], combined with an increasing number of people in the higher age groups [4], may eventually lead to substantially more persons suffering a stroke or having residual symptoms from previous stroke. Optimal rehabilitation is therefore important both for the individual stroke patient and regarding societal costs caused by stroke.

Early supported discharge (ESD) is a rehabilitation concept emphasising early discharge from institution to home for patients after stroke, followed by rehabilitation while home-dwelling, and often supervised by a specialised multi-disciplinary team [5]. This has been studied in several randomised controlled trials previously and the results have been analysed in an updated Cochrane report from 2012 [5]. This meta-analysis suggested a beneficial effect of ESD for a selected group of stroke patients, but at the same time pointed towards the need for future studies in order to clarify which elements of ESD in the primary health care were important, and for more precise clarification of cost-benefit for different patient groups. The results from eleven trials where rehabilitation was offered either centre-based or home-based were reviewed in a recent meta-analysis, and the authors found a significant effect in favour of home-based rehabilitation at 6 weeks and 3–6 months [6]. An international consensus document was recently published with statements regarding team composition, model of team work, intervention, and success [7].

The present ESD Stroke Bergen study was established in order to compare the rehabilitation results after ESD to rehabilitation as usual and to investigate the effect of community treatment given in two different settings; either in a day unit (ESD 1) or in the patients’ homes (ESD 2). We therefore designed a randomised controlled trial with two different ESD arms and one control arm, and with two null hypotheses: (1) ESD does not improve functional outcome evaluated by mRS; and (2) day unit and home rehabilitation are equally effective.

## Methods

### Study design

The ESD Stroke Bergen study is an open randomised controlled trial with three study arms, two for ESD and one control arm. The study was conducted in collaboration between the Department of Physical Medicine and Rehabilitation (DPMR) and the Department of Neurology at Haukeland University Hospital, the University of Bergen (Department of Global Public Health and Primary Care; Physiotherapy and Lifestyle Epidemiology Research Groups) and the Municipality of Bergen, Norway. The full protocol for the study has been published previously and should be consulted for additional details [8]. The study was approved by the Western Norway Regional Committee for Medical Research Ethics and registered in ClinicalTrials.gov with registration number NCT00771771.

### Patient inclusion and randomisation

All patients admitted to the stroke unit at the Department of Neurology, Haukeland University Hospital with suspected stroke were screened for inclusion. Inclusion criteria were living at home in the Municipality of Bergen prior to having a stroke, stroke within the previous seven days, being admitted to the stroke unit within the previous five days, and a National Institutes of Health Stroke Scale (NIHSS) score of 2–26. The scale used in this study was a 13 items Norwegian version (range 0–34) [9]. Patients with NIHSS score <2 were included if modified Rankin Scale (mRS) [10] score was ≥2. The patients had to be awake and able to agree to participation in the study by signing an informed consent, either themselves or by their relatives. There were no age limits. Exclusion criteria were serious psychiatric disorders, alcohol or substance abuse, other serious conditions of importance to the cerebral disorder or subsequent rehabilitation process, or poor knowledge of the Norwegian language.

Patients found eligible by a stroke physician were informed by a designated nurse and asked to participate. Participants were randomised according to a computer-generated block randomisation list (six patients in each block; two for each study arm) and consecutively assigned to their groups in the same order as they were included into the study. The randomisation list was kept by a study coordinator and was not known to any persons in the stroke unit. The inclusion period lasted from 8 December 2008 to 20 December 2011, and 306 patients fulfilling the pre-specified criteria were included.

### Description of study arms

Patients in two of the three study arms were treated according to the ESD concept. They were followed by a designated multi-disciplinary ambulatory team consisting of a nurse, a physiotherapist and an occupational therapist from soon after admission to the stroke unit until shortly after discharge to home. This team originated from DPMR and served as a coordinating link between the patient, relatives, hospital personnel and the personnel in the primary health care. The team was particularly important in the discharge process and cooperated closely with the municipal health care in the planning and implementation of further treatment after discharge.

The two ESD arms differed by the location of treatment: patients in the ESD 1 group received their treatment in a community day unit, whereas ESD 2 group patients stayed in their homes with home-visits from the community health team. Patients in the third study arm constituted a control group and were treated as usual without any intervention from the study, except appointments for testing at DPMR at 3 and 6 months. The treatment “as usual” mainly comprised institutional stay if necessary and/or physiotherapy as needed in the municipality (0–2 hours per week). Patients in all three study arms received language therapy as needed, regardless of allocated arm.

The patients in the two ESD arms were discharged to their homes as soon as possible. Patients in need of a longer in-patient treatment period than offered by the stroke unit were discharged to a municipal institution or DPMR for a period before going home. All patients in the ESD arms were offered rehabilitative treatment by a multi-disciplinary community health team, consisting of a nurse, a physiotherapist and an occupational therapist. The scheduled treatment period was five weeks and maximally four hours per day five days a week, but many patients did not comply with this. Reasons included tiredness, low capacity and lack of motivation, as well as many patients being only minimally neurologically affected and therefore not needing this therapy.

During the treatment period one or more persons from the community health team were present three days a week, and the last two the days of the week the patients trained by themselves after instructions from the team. Part of our study population was also studied by Gjelsvik et al. regarding physical tests and particularly balance, and in this subgroup mean total treatment time for the three days per week supervised by the community health team was 22 hours for ESD 1 and 16.6 hours for ESD 2 patients [11]. After the five week period ESD patients were strongly encouraged to continue training and social activities. They were also offered out-patient 3- and 6-months follow-ups at DPMR with a physician together with a member of the DPMR multi-disciplinary ambulatory team.

### Study outcomes and test procedures

The primary study outcome was mRS at 6 months. Secondary outcomes included mRS at 3 months, as well as NIHSS, Barthel ADL Index (BI) [12] and patient satisfaction (five-point Likert scale with 1 best) at 3 and 6 months. Baseline tests were done before study arm allocation and the individual patient’s group was therefore not known to the patients or the baseline testers. Also at 3 and 6 months the testers were blinded for study arm and the patients were instructed not to reveal this information.

Background and clinical information relevant to the study were prospectively registered in a database (The Bergen NORSTROKE Registry) [13]. NIHSS was assessed immediately after admission and repeated regularly during the first week. The last NIHSS score recorded during the acute phase was used for the baseline analyses. mRS and BI were assessed on day 7, or earlier if the patient left the stroke unit sooner. Participants were re-tested at 3 and 6 months after inclusion at the DPMR out-patient clinic, and for the ESD 1 and 2 groups coupled to the corresponding follow-up. Control group patients were not followed-up by the study physician and advised to contact their general practitioner if they had medical questions of any kind. For various reasons some patients did not want to travel to the DPMR out-patient clinic for testing, and in these cases NIHSS, mRS, BI and patient satisfaction were scored by the testers coming home to the patients if permitted.

To look for possible differences between the study arms, information on in-patient days in hospital or municipal institutions during the first six months after inclusion was collected for all participants.

### Statistical methods and sample size

At study start at least 400 included patients were anticipated, but that had been revised to 350 when the study’s protocol was published [8]. This sample size of approximately 350 included patients was divided into two intervention arms and a control arm, each of 117 patients. Based on a previous relevant study of acute stroke patients [14] the expected proportions of patients with mRS ≤2 at 6 months were 65% for the ESD groups and 51.9% for the control group. This provided a power of 73% (one-sided analysis) to demonstrate a statistically significant difference between the groups.

Demographics were analysed by analysis of variance (ANOVA) (age) and χ^2^-test (gender). Outcome differences between the groups at 3 and 6 months and within-group differences were analysed by nonparametric ANOVA using the Kruskal-Wallis test, and by χ^2^-test for dichotomised outcomes. Discharge destination and days in institution were analysed by χ^2^-test and Kruskal-Wallis test. All analyses were done as intention-to-treat and no imputation of missing data was made. The statistical programmes package Stata/SE 13.1 for Windows (StataCorp LP, Texas 77845, USA) was used for all data analysis.

## Results

**Figure 1:**
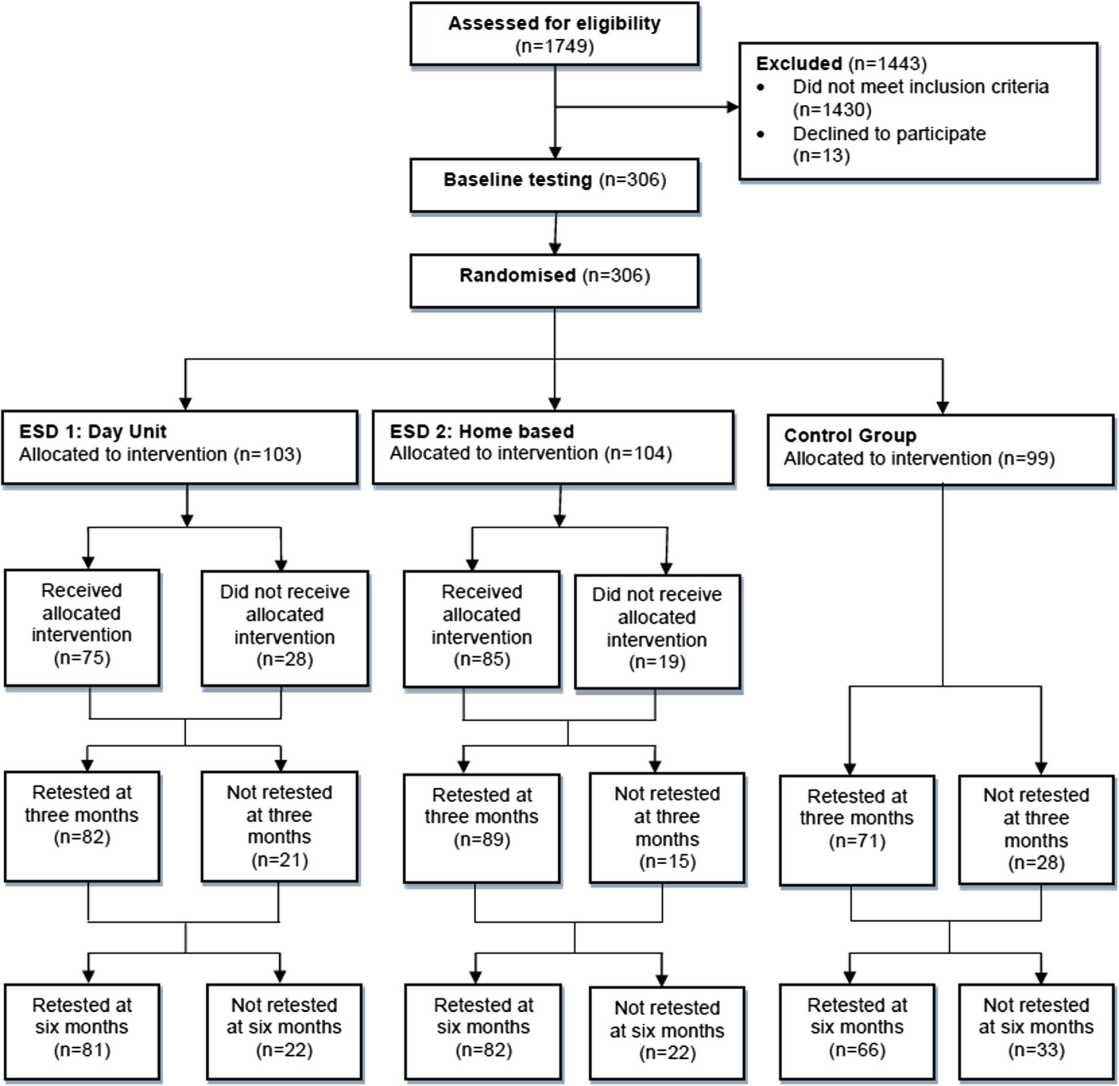
**CONSORT flow-diagram showing patient flow from initial assessment for inclusion to six months testing in the Early supported discharge after stroke in Bergen study.**

1749 patients were screened for participation and 306 were finally included (Figure 1) reducing the power described above to 68%. The main reasons for exclusion were not living in Bergen (approximately 50%) or not having suffered a confirmed stroke (approximately 25%), whereas only 13 eligible patients declined to participate. Seventy-three and 82% of patients in ESD groups 1 and 2, respectively, received the allocated treatment. The patients’ main reasons for not receiving the treatment were being admitted to long-term municipal institution or declining because they did not want or did not need the allocated treatment. The drop-out rates at retest after 3/6 months were 20%/21% in the ESD 1 group and 14%/21% in ESD 2 group, versus 28%/33% in the control group (Figure 1).

The participants were 55.2% men (mean age 69.6 years) and 44.8% women (mean age 75.6 years). Age and gender were not significantly different between the three study groups (Table 1) and neither were baseline scores for mRS, BI and NIHSS (Table 2).

**Table 1:** **Baseline characteristics of 306 ESD Stroke Bergen study patients**

	**All**		**ESD 1 group**		**ESD 2 group**		**Control group**		
**Variables**	**N = 306**		**n = 103**		**n = 104**		**n = 99**		p-value*
Age; mean (range)									
All	72.24	(27–98)	70.61	(29–91)	72.00	(27–92)	74.19	(32–98)	0.152
Male	69.55	(27–92)	69.27	(29–91)	69.41	(27–92)	70.02	(32–92)	0.947
Female	75.56	(38–98)	72.21	(38–90)	75.67	(44–92)	78.81	(44–98)	0.055
Gender; n (%)									
Male	169	(55.2)	56	(54.4)	61	(58.7)	52	(52.5)	0.665
Female	137	(44.8)	47	(45.6)	43	(41.3)	47	(47.5)	

*Abbreviation*: *ESD* Early Supported Discharge.

*Age analysed by ANOVA; gender analysed by χ2-test.

**Table 2:** Descriptive statistics of modified Rankin Scale (mRS), Barthel Index (BI) and National Institutes of Health Stroke Scale (NIHSS)* at baseline, 3 and 6 months follow-ups and of patient satisfaction at 3 and 6 months in 306 ESD Stroke Bergen study patients

**Variables**	**All groups**			**ESD 1 group**			**ESD 2 group**			**Control group**			**p-value†**
mRS; mean (SD) (n)													
Baseline; all	2.59	(1.22)	(306)	2.52	(1.29)	(103)	2.59	(1.15)	(104)	2.66	(1.23)	(99)	0.711
Males	2.53	(1.22)	(169)	2.52	(1.24)	(56)	2.51	(1.16)	(61)	2.56	(1.27)	(52)	0.959
Females	2.66	(1.23)	(137)	2.53	(1.36)	(47)	2.70	(1.12)	(43)	2.77	(1.18)	(47)	0.651
3 months; all	2.45	(1.41)	(242)	2.45	(1.46)	(82)	2.30	(1.37)	(89)	2.62	(1.40)	(71)	0.316
Males	#2.25	(1.34)	(134)	2.32	(1.44)	(44)	2.08	(1.16)	(51)	2.38	(1.44)	(39)	0.612
Females	2.69	(1.46)	(108)	2.61	(1.48)	(38)	2.61	(1.57)	(38)	2.91	(1.30)	(32)	0.530
6 months; all	2.52	(1.50)	(229)	2.40	(1.53)	(81)	2.46	(1.45)	(82)	2.73	(1.52)	(66)	0.410
Males	§2.26	(1.42)	(122)	2.17	(1.58)	(42)	||2.09	(1.18)	(45)	2.60	(1.50)	(35)	0.248
Females	2.80	(1.54)	(107)	2.64	(1.46)	(39)	2.92	(1.62)	(37)	2.87	(1.57)	(31)	0.796
Change scores 0–3 months	−0.22	(1.08)	(242)	−0.26	(0.93)	(82)	−0.36	(1.21)	(89)	0.00	(1.07)	(71)	0.063
Change scores 0–6 months	−0.15	(1.21)	(229)	−0.23	(1.08)	(81)	−0.36	(1.21)	(89)	0.00	(1.19)	(66)	0.532
Barthel Index; median (IQR) (n)													
Baseline	95	(40)	(304)	100	(50)	(102)	92.5	(35)	(104)	95	(45)	(98)	0.742
3 months	100	(15)	(231)	100	(15)	(78)	97.5	(10)	(86)	100	(20)	(67)	0.976
6 months	100	(15)	(213)	100	(15)	(76)	100	(10)	(77)	100	(15)	(60)	0.977
Change scores 0–3 months	0	(20)	(229)	0	(20)	(77)	0	(20)	(86)	0	(15)	(66)	0.642
Change scores 0–6 months	0	(20)	(211)	0	(20)	(75)	0	(20)	(77)	0	(20)	(59)	0.912
NIHSS; median (IQR) (n)													
Baseline	3	(4)	(304)	2	(4)	(103)	3	(3)	(104)	2	(4)	(97)	0.593
3 months	2	(3)	(226)	2	(3)	(76)	2	(3.5)	(84)	2	(3)	(66)	0.925
6 months	2	(3)	(205)	1	(3)	(73)	1.5	(3.5)	(76)	2	(3.5)	(56)	0.718
Change scores 0–3 months	−1	(3)	(224)	−1	(3)	(76)	−1	(3)	(84)	0	(3)	(64)	0.370
Change scores 0–6 months	−1	(3)	(203)	−1	(3)	(73)	−1	(2)	(76)	−1	(3)	(54)	0.615
Patient satisfaction; mean (SD) (n)													
3 months	1.50	(0.99)	(187)	1.32	(0.78)	(62)	1.49	(0.91)	(70)	1.71	(1.23)	(55)	0.115
6 months	1.59	(1.08)	(197)	1.62	(1.22)	(73)	1.51	(0.98)	(71)	1.68	(1.01)	(53)	0.355

*Abbreviations*: *ESD* Early Supported Dischargem, *SD* Standard Deviation, *IQR* Interquartile Range.

*mRS range from 0 (best) to 6; BI range from 0 to 100 (best); NIHSS range from 0 (best) to 34; patient satisfaction range from 1 (best) to 5.

†All analyses: Kruskal-Wallis test; #Males better than females, p = 0.006; §Males better than females, p = 0.004; ||Males better than females, p = 0.011.

The groups did not differ significantly at any point in time for mean values of the primary outcome mRS, nor for BI and NIHSS scores as well as all change scores (Table 2). However, the two intervention groups generally showed somewhat better scores than the control group. Within-group analyses from baseline to 3/6 months showed significant improvement for the ESD 1 (p = 0.042) and ESD 2 groups (p = 0.001) from baseline to 3 months and a trend for improvement from baseline to 6 months, while there was no significant within-group difference for the control group.

Separate analyses for men and women demonstrated a significantly better mRS mean score in men than in women in all groups together at 3 (p = 0.006) and 6 months (p = 0.004), as well as in ESD group 2 separately at 6 months (p = 0.011). Patient satisfaction was rated better in ESD groups 1 and 2 at 3 months (1.32 and 1.49) compared to the control group (1.71), though not significantly (p = 0.115).

mRS and BI scores dichotomised to independency (mRS ≤ 2; BI ≥ 95) or dependency (mRS > 2; BI < 95) demonstrated non-significantly better results for mRS in the intervention groups pooled together vs. the control group (p = 0.086 at 3 months; p = 0.122 at 6 months) (Table 3). Mean mRS scores were not significantly different between the pooled intervention groups and the control group at 3 (p = 0.204) and 6 months (p = 0.188). Change score for mRS at 3 months was significantly better in the pooled ESD groups than in the control group (p = 0.049), but with no difference at 6 months (p = 0.319) (Table 3).

**Table 3:** **Percentage of patients independent (BI ≥ 95 or mRS ≤ 2) and dependent (BI < 95 or mRS > 2) at baseline and after 3 and 6 months in the ESD arms combined and in the control arm; mRS and mRS change scores at 3 and 6 months in 306 ESD Stroke Bergen study patients**

**Time**	**ESD arms (1 + 2)**	**Control arm**	p-value*
Baseline			
BI ≥ 95; n/N (%)	108/206 (52.4)	51/98 (52.0)	0.950
mRS ≤ 2; n/N (%)	109/207 (52.7)	49/99 (49.5)	0.605
3 months			
BI ≥ 95; n/N (%)	113/164 (68.9)	42/67 (62.7)	0.362
mRS ≤ 2; n/N (%)	93/171 (54.4)	30/71 (42.3)	0.086
mRS; mean (SD)	2.37 (1.41)	2.62 (1.40)	0.204
mRS change score 0–3 months; mean (SD)	−0.31 (1.08)	0 (1.07)	**0.049**
6 months			
BI ≥ 95; n/N (%)	111/153 (72.5)	40/60 (66.7)	0.395
mRS ≤ 2; n/N (%)	90/163 (55.2)	29/66 (43.9)	0.122
mRS; mean (SD)	2.43 (1.49)	2.73 (1.52)	0.188
mRS change score 0–6 months; mean (SD)	−0.21 (1.22)	0 (1.19)	0.319

*Abbreviations*: *BI* Barthel Index, *mRS* Modified Rankin Scale, *ESD* Early Supported Discharge, *SD* Standard Deviation.

*Statistical tests: χ2-test (analyses of dichotomised BI and mRS) and Mann–Whitney U-test (mRS and mRS change score), p-value <0.05 indicated in bold.

Subgroup analysis according to discharge destination from the stroke unit (directly home [n = 171]; to municipal institution [n = 78]; to DPMR [n = 47]; others/died [n = 10, results not shown]) showed significantly poorer mRS at 3 and 6 months for patients in the control group discharged to DPMR (mean mRS = 3.25/3.25) compared to the ESD 1 group (mean mRS = 2.76/2.50) and ESD 2 group (mean mRS = 2.33/2.21) (p = 0.041/0.037). Change scores were, however, not significantly different between the groups for 0–3 and 0–6 months. There were no differences in mRS or mRS change scores between the groups for patients discharged home or to a municipal institution (Table 4). In addition, the patients were divided into three groups as equally sized as possible, according to last baseline NIHSS score (0–1 [n = 88]; 2–4 [n = 130]; ≥5 [n = 86]), and mRS scores were analysed for differences between these groups. There were some significant differences as seen from Table 5, but they were not clinically meaningful.

**Table 4:** **Subgroup analyses: modified Rankin Scale (mRS) at baseline, 3 and 6 months and mRS change scores, according to primary discharge destination in 306 ESD Stroke Bergen study patients**

**Discharge destination**	**ESD 1 group**			**ESD 2 group**			**Control group**			p-value*
Home (n=171); mean (SD) (n)										
Baseline	1.72	(0.88)	(54)	1.97	(0.94)	(60)	1.89	(0.88)	(57)	0.359
3 months	1.69	(1.15)	(39)	1.74	(1.16)	(50)	1.93	(1.00)	(44)	0.461
6 months	1.66	(1.15)	(41)	2.00	(1.30)	(45)	1.94	(1.15)	(36)	0.514
Change score 0–3 months	−0.18	(0.94)	(39)	−0.26	(1.23)	(50)	0.07	(1.09)	(44)	0.252
Change score 0–6 months	−0.10	(1.04)	(41)	−0.05	(1.33)	(45)	0.06	(1.24)	(36)	0.820
Municipal Institution (n = 78); mean (SD) (n)										
Baseline	3.77	(0.75)	(22)	3.56	(0.87)	(25)	3.65	(0.84)	(31)	0.588
3 months	3.50	(1.19)	(20)	3.35	(1.30)	(23)	3.83	(1.29)	(18)	0.521
6 months	3.60	(1.19)	(20)	3.41	(1.44)	(22)	3.71	(1.52)	(21)	0.858
Change score 0–3 months	−0.25	(0.79)	(20)	−0.30	(1.18)	(23)	0.00	(1.19)	(18)	0.509
Change score 0–6 months	−0.15	(0.81)	(20)	−0.05	(1.46)	(22)	0.05	(1.28)	(21)	0.881
DPMR (n = 47); mean (SD) (n)										
Baseline	3.26	(1.05)	(19)	3.22	(0.73)	(18)	3.70	(0.67)	(10)	0.378
3 months	2.76	(0.97)	(17)	2.33	(0.72)	(15)	3.25	(0.71)	(8)	**0.041**
6 months	2.50	(1.29)	(14)	2.21	(0.89)	(14)	3.25	(0.71)	(8)	**0.037**
Change score 0–3 months	−0.41	(1.00)	(17)	−0.93	(0.96)	(15)	−0.50	(0.53)	(8)	0.258
Change score 0–6 months	−0.79	(1.37)	(14)	−1.00	(0.88)	(14)	−0.50	(0.53)	(8)	0.492

*Abbreviations*: *ESD* Early Supported Discharge; *SD* standard deviation, *DPMR* Department of Physical Medicine and Rehabilitation.

*All analyses: Kruskal-Wallis test, p-values <0.05 indicated in bold.

**Table 5:** **Subgroup analyses: modified Rankin Scale (mRS) at baseline, 3 and 6 months and mRS change scores, according to baseline NIHSS score in 306 ESD Stroke Bergen study patients**

**Baseline NIHSS score**	**ESD 1 group**			**ESD 2 group**			**Control group**			p-value*
NIHSS 0–1 (n = 88); mean (SD) (n)										
Baseline	1.64	(0.99)	(28)	1.96	(1.02)	(25)	1.83	(1.12)	(35)	0.561
3 months	1.45	(0.96)	(22)	2.05	(0.95)	(22)	1.96	(1.15)	(26)	0.109
6 months	1.32	(1.09)	(22)	2.25	(0.79)	(20)	2.00	(1.65)	(20)	**0.016**
Change score 0–3 months	−0.36	(0.85)	(22)	0.05	(1.13)	(22)	0.23	(1.34)	(26)	0.187
Change score 0–6 months	−0.27	(1.08)	(22)	0.20	(1.11)	(20)	0.20	(1.67)	(20)	0.424
NIHSS 2–4 (n = 130); mean (SD) (n)										
Baseline	2.35	(1.06)	(48)	2.17	(0.93)	(46)	2.50	(0.85)	(36)	0.424
3 months	2.32	(1.34)	(34)	1.74	(1.33)	(38)	2.09	(0.90)	(23)	0.059
6 months	2.40	(1.33)	(35)	1.83	(1.42)	(35)	2.25	(0.99)	(24)	**0.049**
Change score 0–3 months	−0.18	(1.00)	(34)	−0.47	(1.22)	(38)	−0.22	(0.80)	(23)	0.202
Change score 0–6 months	−0.11	(0.99)	(35)	−0.37	(1.44)	(35)	−0.08	(0.83)	(24)	0.238
NIHSS ≥5 (n = 86); mean (SD) (n)										
Baseline	3.74	(1.02)	(27)	3.64	(0.74)	(33)	3.88	(0.71)	(26)	0.391
3 months	3.46	(1.33)	(26)	3.24	(1.21)	(29)	4.00	(1.21)	(20)	0.114
6 months	3.38	(1.53)	(24)	3.44	(1.37)	(27)	3.95	(1.23)	(20)	0.411
Change score 0–3 months	−0.27	(0.92)	(26)	−0.52	(1.21)	(29)	0.00	(0.97)	(20)	0.168
Change score 0–6 months	−0.38	(1.21)	(24)	−0.22	(1.40)	(27)	−0.05	(1.05)	(20)	0.813

*Abbreviations*: *NIHSS* National Institutes of Health Stroke Scale, *ESD* Early Supported Discharge, *SD* standard deviation.

*All analyses: Kruskal-Wallis test, p-values <0.05 indicated in bold.

*Post hoc* we analysed for group differences in discharge destination from the stroke unit, length of continuous stay in hospital or municipal institution after the stroke as well as total days spent in the stroke unit, DPMR and municipal institution during the first six months after stroke. There were no differences between the groups (Table 6).

**Table 6:** **Discharge destination and days of in-patient treatment first six months after inclusion in 306 ESD Stroke Bergen study patients**

**Variables**	**All N = 306**	**ESD 1 group n = 103**	**ESD 2 group n = 104**	**Control group n = 99**	p-value*
Discharged from stroke unit to home; n (%)	171 (55.9)	54 (52.4)	60 (57.7)	57 (57.6)	0.687
Days in institution from stroke to first discharge home within first 6 months after inclusion; mean (SEM)	38.4 (2.9)	37.7 (5.1)	35.6 (4.6)	42.2 (5.6)	0.881
Total days in institution from stroke until 6 months after inclusion; mean (SEM)	45.5 (3.1)	46.0 (5.5)	42.8 (5.0)	47.7 (5.5)	0.930
Days in stroke unit	11.4 (0.4)	11.3 (0.6)	11.3 (0.7)	11.6 (0.8)	0.919
Days in DPMR	7.3 (1.2)	7.9 (2.0)	7.6 (1.9)	6.5 (2.3)	0.560
Days in municipal institution	24.0 (2.6)	23.4 (4.6)	21.5 (4.5)	27.4 (4.5)	0.111
Other	2.7 (0.4)	3.4 (0.7)	2.5 (0.5)	2.1 (0.6)	0.074

*Abbreviations*: *ESD* = Early Supported Discharge; *SEM* = standard error of the mean; *DPMR* = Department of Physical Medicine and Rehabilitation.

*Analysed by χ2-test (discharge to home) and Kruskal-Wallis test (institution days).

## Discussion

The primary research aim was to investigate whether two different ESD regimens were superior to treatment as usual. We found, in general, small differences in favour of the ESD groups for the outcomes mRS, NIHSS, BI, relevant change scores and patient satisfaction when examined by ANOVA, but mostly not statistically significant.

We think that the main reason for these rather small differences in rehabilitation outcome between the early supported groups and the treatment as usual group must be ascribed to the quite different and superior treatment of stroke patients today, as compared to 10–15 years ago when most previous studies were carried out. Stroke prophylaxis is much improved, as well as acute stroke treatment with thrombolysis being used in more than 20% of stroke patients in our region. In addition, the rehabilitation services in general have improved during this period, making it difficult to demonstrate further improvement without quite large study groups.

There is also a possibility that the outcomes mRS and BI, which are in the activities domain of the ICF, are too blunt to detect improvements in more specific physical functions or more qualitative outcome differences between the ESD and control treatment, e.g. regarding quality of life. Gjelsvik et al. reported the results of physical tests in a subgroup of our patients that was discharged directly from the stroke unit to their homes and found statistically significant differences between the groups for trunk control, self-report on walking and ADL [11].

The power calculation for the study was based on the results from a previous large Norwegian study carried out in Trondheim during the period 1995–1998 [14]. In this study the outcomes mRS and BI were dichotomised to dependency and independency and there was a substantial difference at 6 months (p = 0.017 for mRS; p = 0.056 for BI) between patients subjected to early supported discharge and controls. Since comparison between ESD and non-ESD models was a main objective in our study, some of our analyses were repeated with the ESD 1 and 2 groups pooled together vs. the treatment as usual group and the outcomes mRS and BI dichotomised (Table 3). These analyses demonstrated a non-significantly better result for mRS in the two ESD groups compared to the controls (p = 0.086 at 3 months; p = 0.122 at 6 months). The corresponding mRS change score difference at 3 months was marginally significant (p = 0.049). There were no differences for BI.

In retrospect, such a large difference as in the Trondheim study could not be realistically expected in our patients now compared to their patients 15 years ago, due to the improved acute stroke treatment and pharmacological prophylaxis of today. Our study may therefore be considered as underpowered. We also did not reach neither the initially intended number of at least 400 included patients nor the revised number of 350, thereby further reducing study power. However, we recruited almost all eligible patients during the study period and a longer recruitment period was not realistically feasible.

We performed subgroup analyses according to the severity of the initial neurological affection, based on the suggestion that ESD models are assumed most beneficial to stroke patients with a medium severe neurological affection [5]. The patients were divided into three groups according to last baseline NIHSS score, and mostly the three study arms improved about equally in these three NIHSS subgroups (Table 5). There were, however, some statistically significant differences between mRS scores that were clinically inconsistent and not accompanied by significant differences in corresponding change scores, and therefore considered to be without relevance.

We also analysed the primary outcome mRS according to whether the patients were discharged directly to home, to a municipal institution, to DPMR or otherwise (other specialised institution or died in hospital). For patients discharged to DPMR before going home there was a significant difference with the ESD 1 and ESD 2 groups scoring better at 3 and 6 months compared to the control group. The corresponding change scores were on the other hand not significantly different and the clinical significance therefore unclear. There were no differences between the groups for the other discharge destinations.

*Post-hoc* we also analysed the in-patient days for patients in the three study arms, but there were no statistically significant differences in this respect as seen from Table 6. Quite unexpectedly, there also was no difference between the two ESD arms and the control group for length of stay in the stroke unit (11.3, 11.3, and 11.6 days respectively). This demonstrates that today, at least in our region, all stroke patients are discharged from the stroke unit as soon as possible and there is therefore not more to gain in this respect. In fact, the determining factor for the patients’ length of stay in this study was predominantly their need for institutional stay after the discharge from the stroke unit. Patients going directly home spent 8.3 days in the stroke unit whereas those needing an institutional bed after discharge stayed for 15.4 days, with only minute differences between study arms (results not shown).

The other main research question was whether the rehabilitation setting (day unit or home) was of importance for the clinical outcome, but we found no differences between the groups. Hillier et al. have previously reported on centre-based vs. home-based models in a meta-analysis from 2010 comprising 11 trials [6]. They found home-based treatment to be superior as measured by BI in the early period post-discharge, while the results at 6 months were conflicting. They concluded that the rehabilitation should be shifted towards more home-based services and specifically recommended that client preference should be used to determine treatment location in individual patients [6].

We found that men made a better recovery than women in the two intervention groups as judged by mRS. Similar findings have been reported repeatedly. Paolucci et al. reported in a case–control study from 2006 that men and women had a similar neurological recovery, whereas functional recovery was better in men [15]. Wyller et al. reported from Norway in 1997 that women seemed to be functionally more impaired than men [16]. Kim et al. in 2010 reported a similar difference [17]. A possible reason for men recovering better than women in our study may be a higher degree of social support, since substantially more women were living alone (results not shown).

Besides suboptimal power a major challenge in this study was to obtain enduring compliance of included patients during the study period. They were generally old and many found travelling to the out-patient clinic demanding, necessitating testing in their homes. On this background we regard the follow-up rate in our study of about 80-85% for the two intervention arms as satisfactory. The figure of about 70% in the treatment as usual group may be explained by these patients feeling more weakly connected and committed to the study since they did not receive any treatment or medical follow-up from the project.

This study has several clinical implications. Due to the future increase of people in high age groups and consequently increased anticipated disease burden, treatment and rehabilitation models outside specialist health care are strongly needed. To our best knowledge this is the first large randomised controlled trial examining ESD after acute stroke since the pivotal studies before and around 2000. The results indicate a slightly better improvement in the ESD groups compared to controls despite a generally much improved stroke treatment. On the other hand, the length of stay has already been cut strongly down making it difficult to further reduce in-patient time.

Concurrently with the planning of the present study the Municipality of Bergen wanted to develop and strengthen their community rehabilitation, and this enabled a close cooperation between our hospital and the community during the study period. This collaboration has led to a permanently improved rehabilitation service in our community which will benefit patients beyond the stroke category. The final model for ESD rehabilitation, and possibly rehabilitation outside of institution in general, should probably not be a question of treatment either at home or in a day unit, but rather a combination based on clinical judgement by the community health team for the individual patient. This was also suggested by Hillier et al. [6]. In the Municipality of Bergen this model has now been implemented after the study period ended. Another constraint imposed by the RCT study was the duration of the community health team’s treatment period, which has also been modified after study completion and is now upwards limited to three months. There are, however, still improvements to be made. A recent qualitative study by Taule et al. [18] where eight stroke patients from the ESD 2 (home) group were interviewed indicates unmet needs concerning existential and emotional distress after stroke.

## Conclusions

The two ESD groups showed better functional improvement than the treatment as usual group, but not statistically significant. There was no difference between the two ESD groups. The main treatment difference between the intervention groups and the controls turned out to be the supported discharge with later follow-ups, since patients were discharged equally early in all three groups.

Our interpretation: Improved prophylaxis and treatment during the last decade has led to reduced neurological disability after stroke, in parallel with reduced in-hospital time. The added effect of supported discharge and improved follow-up seems to be rather modest and larger patient samples are probably necessary to demonstrate a benefit with statistical significance. In addition, the outcome measure mRS is probably not optimal for measuring meaningful change.
